# Managing human-AI collaborations within Industry 5.0 scenarios via knowledge graphs: key challenges and lessons learned

**DOI:** 10.3389/frai.2024.1247712

**Published:** 2024-11-11

**Authors:** Franz Krause, Heiko Paulheim, Elmar Kiesling, Kabul Kurniawan, Maria Chiara Leva, Hector Diego Estrada-Lugo, Gernot Stübl, Nazim Kemal Üre, Javier Dominguez-Ledo, Maqbool Khan, Pedro Demolder, Hans Gaux, Bernhard Heinzl, Thomas Hoch, Jorge Martinez-Gil, Agastya Silvina, Bernhard A. Moser

**Affiliations:** ^1^The Data and Web Science Group (DWS), University of Mannheim, Mannheim, Germany; ^2^Department of Information Systems and Operations Management, Institute for Data, Process and Knowledge Management, Wirtschaftsuniverstät (Vienna University of Economics and Business), Vienna, Austria; ^3^Austrian Center for Digital Production (ACDP), CDP Center for Digital Production GmbH, Wien, Austria; ^4^Faculty of Sciences and Health, TU Dublin (Technological University Dublin), Dublin, Ireland; ^5^PROFACTOR GmbH, Steyr, Austria; ^6^Department of Artificial Intelligence and Data Engineering, Istanbul Technical University, Istanbul, Türkiye; ^7^IDEKO Research Center, Elgoibar, Spain; ^8^Pak-Austria Fachhochschule: Institute of Applied Sciences and Technology (PAF-IAST), Haripur, Pakistan; ^9^Software Competence Center Hagenberg (SCCH), Hagenberg, Austria; ^10^Timelex BV/SRL, Brussels, Belgium

**Keywords:** knowledge graph, knowledge integration, human-AI collaboration, process modeling, semantic web, Industry 5.0, smart manufacturing, late shaping

## Abstract

In this paper, we discuss technologies and approaches based on Knowledge Graphs (KGs) that enable the management of inline human interventions in AI-assisted manufacturing processes in Industry 5.0 under potentially changing conditions in order to maintain or improve the overall system performance. Whereas KG-based systems are commonly based on a static view with their structure fixed at design time, we argue that the dynamic challenge of inline Human-AI (H-AI) collaboration in industrial settings calls for a late shaping design principle. In contrast to early shaping, which determines the system's behavior at design time in a fine granular manner, late shaping is a coarse-to-fine approach that leaves more space for fine-tuning, adaptation and integration of human intelligence at runtime. In this context we discuss approaches and lessons learned from the European manufacturing project Teaming.AI, https://www.teamingai-project.eu/, addressing general challenges like the modeling of domain expertise with particular focus on vertical knowledge integration, as well as challenges linked to an industrial KG of choice, such as its dynamic population and the late shaping of KG embeddings as the foundation of relational machine learning models which have emerged as an effective tool for exploiting graph-structured data to infer new insights.

## 1 Introduction

Whereas recent developments around Artificial Intelligence (AI) have mostly focused on how it can replace or help humans by giving them “superpowers,” we focus on situations where technology (still) needs the support of knowledgeable humans. Although the degree of automation in industry is steadily increasing, success and effectiveness still largely depend on human intervention in many situations (Hoch et al., 2022). This is also the case with highly automated processes, for example when adaptations or re-configurations have to be made, or when there is little or no data available to solve a particular problem. In this context, a key question that arises is how AI technologies can help facilitate problem analysis and resolution through tight collaboration between humans and AI.

For more than 25 years, the human-computer interaction (HCI) community has proposed principles, guidelines, and strategies for designing user interfaces and interacting with applications that use AI models (Amershi et al., 2019; Xu and Dainoff, 2023; Xu et al., 2023). The HCI guidelines resulting from empirical studies can be seen as requirements on a model-based approach to tackle H-AI collaboration. While such guidelines remain general and typically do not go beyond a phenomenological approach, they do offer a first systematic basis and orientation for further analyses, questions and model building. Amershi et al. (2019) sketches 18 guidelines addressing various phases of operation covering the *initial phase* on informing the user about the system's capabilities and limitations, the phase *during interaction* by postulating to display contextually relevant information to the user's current task and environment, and to ensure the experience is delivered in a way that users would expect, given their social and cultural context. The transition from high-level guidelines to operational computational models is challenging and remains by and large an open research issue. What does currently exist are scattered case studies on specific aspects, such as, e.g., user experience (Li et al., 2022), parallel and continuous modes of interaction beyond of discrete sequential patterns (Wintersberger et al., 2022), trust calibration (Naiseh et al., 2023), and traceability (Schuitemaker and Xu, 2020). Also, the extensive literature on explainable AI can be included in this context. Beyond that there is conceptual work for a model-based approach.

van den Bosch et al. (2019) distinguishes six main modeling problems in achieving effective human-machine interaction and collaboration: (1), a *taxonomy model*, i.e., a shared vocabulary of concepts, (2), a *team model*, i.e., a shared set of work agreements and interdependencies, (3), a *task model*, i.e., knowledge about the regularities between task conditions, actions and outcomes, (4) a *self-model*: knowledge about itself, including its needs, goals, values, capabilities, resources and plans, (5), a *theory-of-mind model*: knowledge of other agent's needs, goals, values, capabilities, resources and plans, and, (6), a *communication model*. In addition, we add, (7), a normative model, to address boundaries and requirements as a result of the application of laws and regulations—such as general ethical requirements, the European General Data Protection Regulation (GDPR), or the upcoming European AI Regulation. The normative challenge consists of the fact that the modeler will have to act within the boundaries imposed by the laws and regulations. Those normative sources frequently impose considerations that the modeler will have to consider from the outset but which may only materialize once the AI is put into practice. The models (1)–(5) are generally neither static nor complete or certain. Rather they are in need of refinement, updates and adaptation over time. As pointed out by Bansal et al. (2019b), updates that boost AI performance can actually degrade team performance. Therefore, adaptation of the self, team, and theory-of-mind models in response to the dynamics and performance context is required to ensure team effectiveness. This dynamics aspect is one of the major challenges for effective H-AI teaming (National Academies of Sciences, Engineering, and Medicine, 2022). In particular the self-model, respectively, the theory-of-mind model, will be partially incomplete and uncertain. Ultimately, it is this incompleteness and uncertainty that needs to be compensated through functionalities to integrate additional context and H-AI collaboration.

In manufacturing processes, there are AI-intrinsic issues due to inadequacies in system design resulting from non-ideal conditions in the real production environment that deviate from the idealized assumptions at the time of engineering. For example, when training a machine learning model for the purpose of predictive maintenance, it is assumed that the sample data are identically distributed. However, there are often data shifts from the training situation to the application scenario that may not be immediately apparent, cf., e.g. Sugiyama and Kawanabe (2012), Bansal et al. (2019a), and Becker and Becker (2021). Ultimately, humans are required to oversee and interpret appropriate monitoring parameters in order to compensate for such deficiencies and initiate countermeasures. Critical phases occur particularly during retrofitting and re-calibration and setup of a new process. In this case, the self-model of the AI system requires correction by the theory-of-mind model of the human collaborator. Vice versa, human factors remain a key challenge in semi-automation due to mental conditions that are difficult to model (e.g., knowledge, training, capabilities, skills, experience, creativity, limited attention, cognitive bias etc.) as well as ergonomics and physical conditions (e.g., safety, manual, fatigue, posture, wellbeing, gesture, musculoskeletal disorder etc.), c.f., e.g., Neumann et al. (2021) and Ma et al. (2023a). Here, the AI system's theory-of-mind model about its human collaborators' states can help to refine the humans' self model, e.g., detecting ergonomic risks. Additionally, adding a human factor introduces new challenges in relation to the data that is used. In Europe, personal information used in training an AI or to recalibrate the output falls under the scope of the GDPR and, more importantly, the upcoming AI Regulation. Moreover, in the upcoming AI Regulation, important requirements have been introduced for providers and deployers of AI systems, in that they need to take specific action to promote AI literacy and are subject to specific transparency obligations.

In the remainder of this paper, we address this dynamics challenge on the design and handling of knowledge graphs posed by the management of inline human interventions in AI-assisted manufacturing processes under potentially changing conditions. In this context, it is a particular challenge to guide humans to assist AI systems in adapting to changing conditions, rather than AI being assisted by humans, which is the standard view of applying AI systems (Peres et al., 2020; Abioye et al., 2021). Specifically, we tackle the question to what extent and how context and human integration is feasible and discuss the main challenges to achieving it.

The paper is structured as follows: Section 2 provides a problem analysis and outline of approaches, which is illustrated by selected use case scenarios in manufacturing. The main part of the paper is then structured along the engineering cycle of building and integrating a human-AI collaborating system in an industrial process, comprising, (1) methodological challenges of H-AI collaboration (Section 3), (2) enabling technologies to address these challenges (Section 4), and (3), sketch of approaches and lessons learning from the European Teaming.AI^1^ project (Section 5).

## 2 Scenarios for human-AI collaboration in Industry 5.0

Whereas Industry 4.0 (I4.0) focuses on design principles such as intelligent, self-organizing Cyber-physical Production Systems (CPPSs) that are flexible and adapt to changing requirements (Vogel-Heuser and Hess, 2016), the introduction of Industry 5.0 has augmented this vision with a strong focus on humans and machines working in symbiosis (Longo et al., 2020). At the intersection of these visions, collaboration becomes particularly important in flexible, self-organizing, and complex CPPSs that integrate innovative processes and technologies in highly automated, but increasingly flexible cells.

In this context we select three use case scenarios addressing different aspects where human assistance is required. So, there is a special need for H-AI collaboration to tackle process steps that are more difficult to automate—such as

- ensuring *human oversight* in sensitive situations where the AI system cannot be sufficiently trusted and where human contextual understanding is required to manage ambiguous settings, see use case 1 described in Section 2.1 and our proposed implementation in Section 6.1;
- the readjustment of machine parameters during production and the associated tasks of machine diagnostics, which require *in-depth expertise* and the reasoning skills of experienced humans, see use case 2 in Section 2.2, respectively Section 6.1;
- *personalized ergonomic assessment* that requires individual feedback from the person concerned to compensate for the AI system's shortcomings in personalizing its inference, see use case 3 in Section 2.3, respectively Section 6.1.

The limited degree of automation of such steps can be attributed to the fact that humans typically rely on experiences, background knowledge, and a richer set of sensory inputs to form a mental model of the situation and causalities than is reflected in documents and procedural guidelines. However, human reasoning is challenged by limited attention and cognitive capacity, which becomes increasingly difficult with increasingly complex interactions and larger amounts of data. AI-based capabilities can help improve situational awareness to enable better understanding and decision making. However, without human oversight or guidance, AI systems struggle to address situations and contingencies for which there is a lack of data and knowledge at the time of deployment.

### 2.1 Scenario 1: human oversight and process management

While industrial CPPSs are designed to operate without human intervention, they still require significant planning and oversight to ensure that they are properly configured and optimized for their intended use. This requires (1) real-time observability and situational awareness at all levels of the processes, (2) prioritizing which changes need to be made urgently and which can be deferred, without creating an intolerable backlog, (3) executing and implementing planned changes, (4) managing and scheduling all recovery testing, and (5) providing audit trails for all compliance requirements. In this context, humans provide crucial decision-making capabilities and management in process orchestration platforms. While automation and AI algorithms handle routine tasks and process execution, human intervention becomes necessary for complex or ambiguous situations that require judgment, creativity, and contextual understanding.

### 2.2 Scenario 2: root cause analysis and machine diagnostics

Analytic tasks such as root cause analyses in machine diagnostics constitute another broad class of tasks where synergistic collaboration between humans and AI agents is particularly helpful. As an example, consider quality management on the shop floor, which requires (1) careful configuration of production resources during initial setup and ramp-up of a production line, (2) configuration and adaptation during setup for a production run and (3) readjustments of machine parameters during production. To navigate the complex space of potential machine configurations and achieve consistent output with the desired quality characteristics, operators currently primarily rely on their experience and intuition to interpret indicators such as types of defects observed as well as their positions on the produced parts to form hypotheses on the causes which then determine the choice of an adequate adjustment procedure.

### 2.3 Scenario 3: ensuring occupational safety and health

Recent reports^2^ show that among the 170 million workers in the EU, three out of five report health issues in terms of *Musculoskeletal disorders* (MSDs). For detail on MSDs see, e.g., Kee (2023) and Govaerts et al. (2021). Industrial companies often design their work places to avoid safety and health related-problems or invite Occupational Safety and Health (OSH) experts who examine the work process. In this procedure the experts monitor a work process from one to several days and perform a manual analysis which results in an improvement report for those responsible—e.g the shift managers. These approaches are both time consuming and monetarily expensive and they are difficult to apply in more flexible production scenarios due to regularly changing products, e.g., in the domain of high-precision machining of large parts. In such scenarios, the work process itself is developed and optimized along with the first parts produced. Workers and shift managers can hold regular improvement meetings on a daily basis, where they discuss the previous shift and how the work process can be improved. In this context, AI systems show their strength in high coverage and non-tiring monitoring, but it is necessary to rely on human knowledge and feedback to correct or resolve ambiguous situations.

## 3 Knowledge modeling and orchestration challenges

In this section, we point out the need for a late design principle for computational models to support collaboration between humans and AI. In this context, we discuss the challenges for knowledge modeling and population, vertical knowledge integration, and team orchestration.

### 3.1 Late Shaping design paradigm challenges

**Figure 1:**
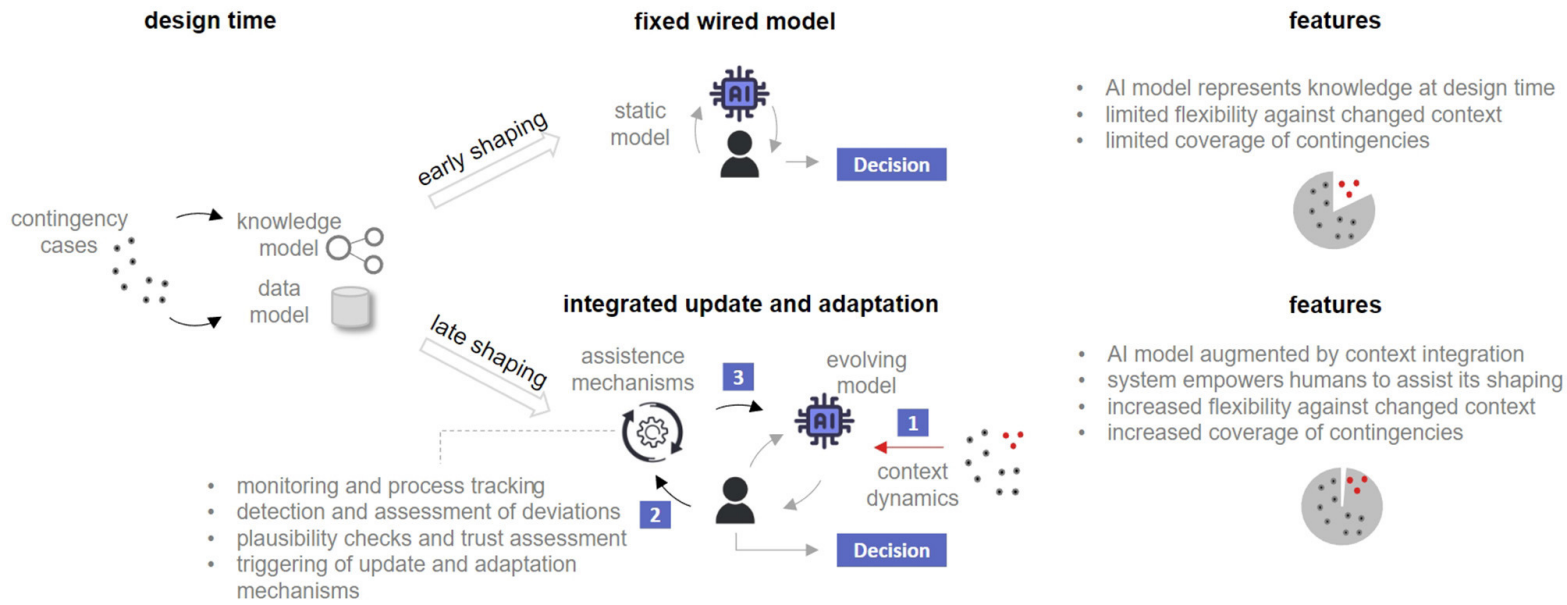
Early vs. late shaping for human AI interaction systems. While *early shaping* fixes the AI model's behavior in a fine granular manner at design time, *late shaping* leaves room for update and model adaptation after deployment. In the latter, human intelligence is used for continuous context integration (1), to assist the system in improving its monitoring and trust assessment (2), and to trigger model updates (3).

In psychology there is the notion of shaping (Peterson, 2004), which reflects the process of strengthening a behavior through punishment and reinforcement. In the setting of AI systems, usually the system's behavior is shaped by training at design time. But as the example of data shift shows, merely early shaping at design time can be too inflexible and insufficient. In contrast to early shaping, late shaping refers to fine tuning in a later phase based on an already trained model, e.g., in a (re-)calibration phase. Early and late shaping is discussed for personalizing machine learning models via transfer learning (Schneider and Vlachos, 2021) and domain adaptation (Dinu et al., 2023) that is to tailor the system's behavior to the characteristics of a sub-sample from the whole universe of samples. In our setting, see Figure 1, we argue that a late-shaping mechanism is needed that goes beyond the purely statistical framework of transfer learning in order to respond flexibly to unforeseen changes for which the original system was not trained. The heterogeneity of data and knowledge representations in our environment goes beyond current machine learning systems, so we are dealing with a hybrid system consisting of machine learning and knowledge models augmented by human intelligence as an additional component. As long as context-aware AI systems are only available for specific sub-domains (Kejriwal, 2021; del Carmen Rodríguez-Hernández and Ilarri, 2021), our approach is to harness human intelligence and experience in the form of background knowledge and common sense to compensate for the shortcomings of current AI systems. From a conceptual perspective, we therefore consider the utilization of context, its supervision and augmentation by humans as key factors for effective H-AI collaboration systems. From a model and system design perspective, the performance of the system improves when it becomes adaptable and can be tailored to the context, thus requiring a late-shaping approach. The advantage of the late shaping approach is that it is possible to make updates and adjustments even after the initial training phase, based on process monitoring and tracking (Ding et al., 2020; Schuitemaker and Xu, 2020), detection and prediction of deviations (Cardoso and Ferreira, 2020), as well as explanations, plausibility checks and trust evaluations (Wang et al., 2022; Naiseh et al., 2023; Ma et al., 2023b) at run time.

### 3.2 Knowledge modeling, population and integration challenges

#### 3.2.1 Knowledge modeling

Knowledge Modeling is a process to identify concepts, entities and their relationship from a given domain in order to conceptualize it. Knowledge modeling plays a crucial role in various domains of H-AI collaboration. It involves the representation and management of different types of knowledge to support effective decision-making and cooperation between humans and AI systems. To this end, it should provide an integration point that connects manufacturing data and knowledge at scale in order to enable the enactment of teaming scenarios on top of this data. The resulting model should create a semantically organized *digital shadow* of the manufacturing environment that enriches and contextualizes events that emanate from the production environment and relates them to relevant domain knowledge.

Specifically, we identified several (potential) areas that the knowledge modeling process needs to cover in the context of H-AI collaboration: (1) *Domain knowledge*: Specific knowledge obtained from specialized disciplines, fields, or business areas (e.g., stakeholders, products, processes, assets, etc.); (2) *Process knowledge*: Knowledge related to business processes aspect such as events, activities, input-output, conditions, etc.; (3) *Policy knowledge*: Regulation, policies, or rules used to control system implementation or application; (4) *Observational knowledge*: Knowledge derived from resources used as the main input of a process, such as sensor measurements, machine parameters, visual tracking events, etc.; (5) *Context knowledge*: Additional knowledge used to contextualize monitoring and decision-making processes, enriching and constraining them based on existing conditions to resolve or mitigate ambiguities; (6) *Teaming knowledge*: Knowledge related to H-AI interaction, encompassing teaming processes, teaming events, and teaming activities. Orthogonal to this, the above categories include both *explicitly asserted* and *inferred knowledge*, i.e., new knowledge that can be deduced from existing knowledge, generated through logical inference, statistical inference, machine learning prediction, etc.

#### 3.2.2 Knowledge population

Knowledge population refers to the process of constructing and maintaining knowledge by integrating diverse data sources and modalities. Knowledge population methods tackle a number of general challenges involved in the construction of a knowledge base—such as, e.g., interoperability, data heterogeneity, quality, and incompleteness.

In particular, we identified the following key challenges for knowledge modeling and population for H-AI collaboration: (1) *Data heterogeneity*: knowledge modeling requires integrating and contextualizing data from heterogeneous source and topic domains. Different sources may have varying formats, structures, and semantics, making it challenging to harmonize and combine them effectively. (2) *Data Scope Management*: determining what data to include in a shared knowledge model is a challenge. Including all available data may lead to issues related to manageability and scalability. Therefore, careful consideration is required to select the most relevant and valuable data for inclusion. (3) *Semantic Localization*: establishing clear mappings between the original data sources and their representations is a challenge. It requires a deep understanding of the domain and expertise in ontology development. (4) *Data Catalog Management*: managing data that does not meet the inclusion criteria, such as large-scale raw data without well-defined semantics poses a challenge. Therefore, it is necessary to ensure that such data is appropriately cataloged and coordinated.

#### 3.2.3 Knowledge integration

To facilitate collaboration between and among human experts from multiple domains and AI agents, knowledge integration based on shared concepts is a key challenge that needs to be addressed. This necessitates a modular, layered approach to (1) deliver a trustworthy, auditable, integrated data store in order to cope with the inherent heterogeneity of manufacturing data, (2) facilitate the combination of different types of AI and human reasoning in an integrated platform, and (3) improve overall decision quality in manufacturing, for example, by allowing for improved parameter selection of injection molding machines.

A number of well-established domain-specific layering models that are prevalent in the manufacturing domain can be used as a foundation for vertical knowledge integration. The ANSI-ISA-95 and IEC 62264 standards, cf. IEC (2003), for instance, partition activities in manufacturing enterprises into a functional hierarchy, i.e., (1) production process, (2) sensing and manipulation, (3) monitoring and supervision, (4) manufacturing operations management, and (5) business planning and logistics. It also introduces terminology and interfaces between these layers and aims to establish a common understanding for all participants and precisely defined interactions between any two layers. More recently, the Reference Architecture Industry 4.0 (RAMI), cf. Adolphs et al. (2015), introduced a three-dimensional conceptual framework that represents the Industry 4.0 space along three axes: (1) life cycle and value stream (IEC 62890), (2) hierarchy levels (IEC 62264), and (3) layers.

### 3.3 Normative constraints

Collecting, labeling, and otherwise using human knowledge to develop AI agents to work together with humans leads to challenges from a normative perspective as well. Two legislative frameworks almost always apply in the European Union to an AI driven teaming context: Regulation 2016/679 on the protection of natural persons with regard to the processing of personal data and on the free movement of such data, otherwise known as the GDPR, and the upcoming AI Regulation (proposal for a regulation on AI).

First, the GDPR is a horizontal regulation, meaning that it applies to any processing of so-called personal data, irrespective of the context or sector. It imposes requirements that must be taken into account by the developer and by any users of AI systems. Notably, (1) they will have to be mindful that certain categories of personal data are deemed sensitive under the GDPR, leading to more stringent legal requirements. For example, in Use Case 3, health data and biometric data is processed of the operators on the shop floor. (2) data can only be processed (incl. collected) in a lawful manner. This means that a legal basis under the GDPR needs to be established before collecting or otherwise processing the data. (3) a transparency obligation applies. Persons whose data is processed need to be informed with respect to the responsible entity, the scope of data collecting, the intended use and recipients of the data, and the planned retention period. (4) they will have to ensure that only relevant personal data is processed and that the data collected and processed is accurate and up-to-date.

Second, the development of AI agents to work together with human actors will be impacted by the upcoming AI Regulation in the future. The proposed AI Regulation distinguishes four risk profiles of AI systems, from standard risk to forbidden risks. The higher the risk of the AI system, the stricter the interpretation of the requirements. Any AI system would require human agency and oversight, with the system being overseen and controlled by humans. A human-AI teaming model suits this criterion quite well. AI systems must also be safe and secured against malicious and unlawful uses by third parties. The AI Regulation furthermore imposes high standards in terms of data quality and integrity, including for training data, and is more demanding than the GDPR with respect to transparency and explainability. Humans need to be made aware whenever they communicate or interact with an AI system, and be informed of the capabilities and limitations of that AI system. These criteria apply more stringently for higher risk systems. For example, the AI system that would be developed under Use Case 3 would be considered a high-risk AI system, whereas the AI systems under the Use Cases 1 and 2 would be qualified as “normal” AI systems.

It is clear that the normative frameworks in the European Union impose significant legal obligations on researchers and users of AI systems. These frameworks are more and more intertwined, and increasingly require developers to have access to both legal and technological insight. European legislation imposes particularly high standards of transparency, proportionality, explainability and human involvement - topics that, as the reader will recognize, teaming models are relatively well suited to addressing.

### 3.4 Human-AI teaming orchestration challenges

In contrast to typical human-in-the-loop settings, teaming orchestration addresses a broader scope of tasks, e.g., to cope with oversight and management problems as outlined in Section 2.1. Teaming orchestration, i.e., monitoring and real-time management of complex activities (Yang et al., 2023; du Boulay et al., 2023) is challenging because it needs to take into account the different and unique characteristics of its participants, but also combine their respective strengths.

Rather than using AI as a mere substitute for humans, teaming orchestration needs to lean into AI factors and leverage the innate abilities of AI systems in order to overcome teaming limitations in high-complexity collaborative tasks (Zhang et al., 2021). For this, orchestration needs to follow a structured approach that is also generalizable across use cases and domains. On the one hand, it requires clear rules to determine, for example, when to request data labels from a human operator, or which samples to choose intelligently to be most effective. AI systems should guide humans and make appropriate queries to obtain the desired information. At the same time, it is important to efficiently manage and use the managed data, both labeled and unlabeled, to improve and evolve an AI system over time. On the other hand, implementing these rules in software necessitates a generalizable approach that allows the creation of workflow pipelines without modifying the software code extensively when adopting the software platform to a new use case or refining (late shaping) existing deployments. To address these challenges, we aim for a model-based approach that allows the specification of teaming workflows using process models that allow changing communication pattern and sequences by means of software configuration, thus ensuring flexibility and maintainability in large-scale application scenarios. Moreover, incorporating the dynamics of the teaming process into these workflows at runtime poses a further challenge. Merely relying on a single static process model is insufficient; instead, we need to consider alternative pathways, dynamic switches between these alternatives, and variations in path executions, such as role sharing. To achieve this, we need to combine process models with dynamic information modeling that captures runtime dynamics in process executions.

Realizing and implementing these coordination mechanisms by means of computational models is still an under-explored research challenge (Bansal et al., 2019a; Zhang et al., 2021).

## 4 Enabling technologies

This section provides an overview of the modeling and representation technologies applied in this work.

### 4.1 Methods for knowledge graph modeling and population

Knowledge Graphs are a powerful tool for knowledge modeling. They provides a comprehensive and interconnected view of knowledge that enables effective knowledge organization, retrieval, and reasoning. The potential of KGs for knowledge modeling lies in their ability to capture and represent rich semantic relationships between entities. Unlike traditional databases or hierarchical taxonomies, KGs allow for flexible and dynamic modeling, which accommodates various types of relationships and complex knowledge structures.

To develop and structure their *schema*, several methodologies can be adapted and reused that have been developed for ontology engineering (Noy and McGuinness, 2001; Fernández-López et al., 1997; Sure et al., 2004; De Nicola and Missikoff, 2016). In our industrial setting, methods for rapid ontology engineering such as Sure et al. (2004); De Nicola and Missikoff (2016) that focus on agile and application-driven development with strong involvement of industrial domain experts are particularly suitable in the context of rapidly changing requirements (c.f. Spoladore and Pessot, 2022 for a review and evaluation).

Beyond the schema-level, *Knowledge Graph construction* approaches can more broadly be characterized along several dimensions: *Manual vs. (semi-) automated*: KGs can be manually constructed or automatically populated. Manual construction involves expert-driven or community-driven approaches but can be costly and manual curation can quickly become infeasible for large-scale KGs (Stepanova et al., 2018). WordNet (Miller, 1995), Freebase (Bollacker et al., 2008), Wikidata (Vrandečić and Krötzsch, 2014) are examples of this approach. Manual construction is also not feasible for continuously updating a KG based on events and observations from a live (e.g., cyber-physical production) system. The automated population uses existing (semi-) structured sources such as YAGO (Suchanek et al., 2007) and DBPedia (Lehmann et al., 2014) or extracts knowledge from unstructured text such as NELL (Carlson et al., 2010). However, this may come with limitations in terms of precision and quality.

### 4.2 Methods for process modeling, monitoring and orchestration

Business processes provide organizations a way to flexibly compose a set of activities that are performed in a defined order to achieve a business goal (Weske and Weske, 2019). These activities can be executed by a human, a machine, another entity (e.g., a software system) or a combination thereof.

Process models enable the formal definition of such processes by describing the structure of the process and assigning units of work to agents that perform the assigned task. These units of work are called process activities. The structure defined by process models may range from a simple sequential order to complex structures that involve different gateways and loops, c.f. Weske and Weske (2019). Dedicated languages to model business processes [such as Business Process Modeling and Notation (BPMN)] have also been adopted in manufacturing scenarios (Prades et al., 2013). Process can be described either declaratively or imperatively. In programming, imperative programming languages imply to “say how to do something” (Van Roy and Haridi, 2004) whereas declarative paradigms “say what is required and let the system determine how to achieve it.” Hence, imperative programming languages describe exactly the control flow of computation whereas declarative programming languages focus on expressing the logic of computation.

Similarly, imperative process modeling is characterized by an inside-to-outside approach (Pichler et al., 2012) that requires that each step of a process—including all possible execution alternatives—is explicitly specified before the process is executed. Declarative process modeling, on the other hand, follows an outside-to-inside approach, i.e., the process is not defined beforehand, but only its essential characteristics are defined as constraints on the model that must not be violated during execution, c.f. Pichler et al. (2012). These constraints are usually exerted over activities and express norms, behavioral patterns and best practices that restrict the possible behavior and execution order of the activities (van Dongen et al., 2021).

With respect to the respective strengths and weaknesses of these modeling paradigms, we found that they are not uniform across our collaborative use cases. Whereas the declarative modeling approach allows more flexible execution and is therefore well-suited for volatile environments (Mazzola et al., 2017), which is beneficial, e.g., for diagnostic tasks such as Use Case Scenario 2, the imperative paradigm has benefits in terms of minimal description length (van Dongen et al., 2021) and human understanding (Haisjackl et al., 2013; Di Ciccio et al., 2019). Furthermore, we found that most of the Use Case Scenarios can easily be described imperatively and that the required flexibility can be reintroduced in imperative modeling environments by decoupling independent and autonomous, but imperatively structured processes through (1) message flows and events, and (2) abstract activities.

### 4.3 Knowledge graph embeddings as enablers of relational machine learning

Industrial KGs can be employed to integrate and standardize domain knowledge. However, the application of KG embeddings as lean KG feature representations of graph elements has yet to be extensively explored in this domain. We utilize the notion of a (standard) KG G=(V,E), which is identified by a set of nodes *V* and a set of triples *E*⊆*V*×*R*×*V* consisting of directed and labeled edges, with *R* denoting the set of valid relations defined in an underlying ontology (Krause et al., 2022). Thus, a KG fact (*s, p, o*)∈*E* denotes an edge from the subject *s*∈*V* to the object *o*∈*V* via the predicate *p*∈*R*. Given a KG and dimension *d*∈ℕ, embedding methods exploit the KG topology to generate feature representations γ:*V* → ℝ^*d*^.

Besides an improved applicability of graph-based data in recommendation systems (Palumbo et al., 2018) or question answering (Diefenbach et al., 2020), KG embeddings have also proven to be valuable as complements to graph data. This is due to their ability to provide an empirical approach for enhancing the expressivity of graph topologies by means of downstream tasks like entity linking (Sun et al., 2018) and link prediction (Rossi et al., 2021). Accordingly, relational ML receives significant attention in both literature and applications (Nickel et al., 2016), building upon the idea of utilizing KG embeddings as lean feature representations to be fed into downstream Machine Learning (ML) models. Besides transfer approaches like RDF2Vec (Ristoski et al., 2019) to derive KG embeddings from random graph walks via NLP methods such as Word2Vec (Mikolov et al., 2013), KG embedding methods can be categorized into geometric models, tensor decomposition models, and deep learning models (Rossi et al., 2021).

To apply KG embedding formalisms within the use cases presented in this paper, a process-oriented KG representation is indispensable to effectively integrate relational machine learning as an enabler of the validation of data consistency and the empirical derivation of new knowledge. Such a KG plays a fundamental role in facilitating the dynamic adaptation of ML models to semantically linked industrial data, ensuring a more robust and reliable system for knowledge extraction and integration. In the following, approaches are proposed to dynamically encode teaming knowledge within a corresponding KG.

## 5 Approaches on modeling and orchestrating human-AI processes

**Figure 2:**
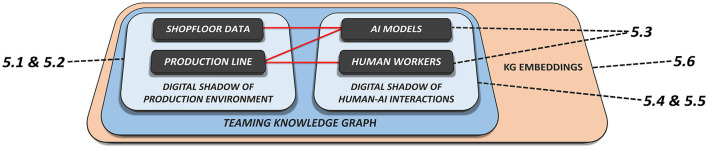
Schematic interconnection of the solution approaches presented in this chapter based on the manufacturing elements and KG components that are addressed in the corresponding subchapters.

This section provides solution approaches that aim to resolve the key challenges introduced in Section 3 regarding the implementation of H-AI interactions within the use case scenarios introduced in Section 2 by leveraging the enabling technologies from Section 4. For a related software architecture we refer to Haindl et al. (2022). Figure 2 provides a comprehensive overview of the following sub-sections, including their thematic connections. Sections 5.1 and 5.2 introduce the modeling and population of industrial KGs as digital shadows of existing manufacturing environments. Section 5.3 addresses the task of orchestrating BPMN Teaming processes from an engineering perspective. Based on these findings, Sections 5.4 and 5.5 provide approaches to integrate BPMN process models and their dynamic executions in a shared KG, linking it to existing domain knowledge. Sample implementations for the previously introduced use case scenarios are described in Section 2. Moreover, the knowledge representations proposed in this chapter can ultimately be adopted in relational ML models based on dynamic KG embeddings which are to be introduced in Section 7, allowing for empirical late shaping approaches.

### 5.1 Vertical knowledge integration

To address knowledge integration challenges in the context of H-AI teaming in manufacturing, we developed a layering approach for H-AI collaboration that focuses on the vertical layer axis in RAMI I4.0 (Adolphs et al., 2015). This axis decomposes complex I4.0 manufacturing systems into loosely coupled layers, adopting principles from information systems and software engineering. We leverage this layering model as a framework for the conceptualization of a KG to support vertical knowledge integration. Mapping concepts to layers facilitates a modularization of the KG that is consistent with the layering of the manufacturing system. For example, vocabularies to express sensor observations are assigned to and used across the lower layers, whereas policy specifications, expert-knowledge-derived decision rules, or process models will be scoped and restricted to the higher layers.

The use case presented in Section 6.2, for instance, is composed of the steps: (1) setup of the injection process, (2) production, and (3) quality inspection. The layering allows to interface with the domain experts appropriately: the process engineer for example acts at the communication and integration layers [typically represented by Supervisory Control and Data Acquisition (SCADA) and/or Programmable Logic Controller (PLC) systems]. At production run time, the layers serve as a guideline to structure the interactions between components and stakeholders with the knowledge graph, based on the modularization principles of the framework. Note that the layering approach does not imply architectural choices or commitment to particular implementation options (for example interfaces, or whether and how the data represented in the knowledge graph is materialized). Classifications made by an automated visual quality inspection components, for instance, are assigned to the integration layer and may be propagated from there through higher-level events to the upper layers. In this context, the knowledge graph represents a partial view on the real-world system that links relevant aspects for a given perspective.

**Figure 3:**
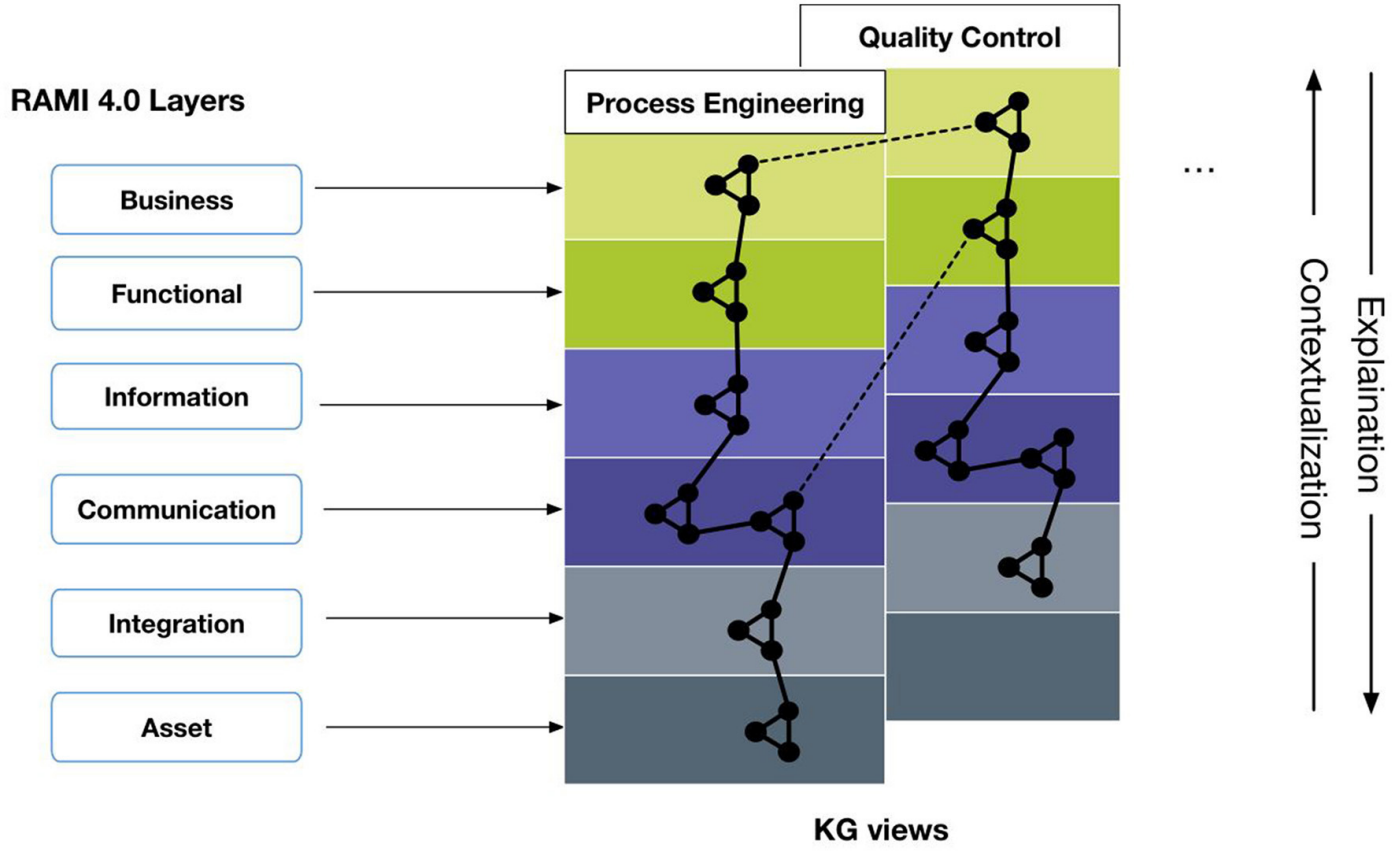
Knowledge graph layering concept.

Methodologically, we propose a modular and incremental approach to develop knowledge graph-based applications in Industry 4.0; rather than developing a single, monolithic KG, which requires a large up-front investment and bears considerable risk, our approach aims to incrementally build a system-wide knowledge graph as an aggregation of more granular per-use case views, while ensuring their conceptual alignment through a set of shared core concepts. This also enables cross-view linking—either through a shared underlying knowledge graph or explicit, user-generated links—and navigation across multiple perspectives (dashed lines in Figure 3).

#### 5.1.1 Lessons learned

The application of a layering approach in H-AI collaboration, particularly within the framework of Industry 4.0 manufacturing systems, offers significant benefits for the conceptualization and development of a knowledge graph aimed at vertical knowledge integration. By mapping distinct concepts to various layers, the knowledge graph becomes modular and is systematically aligned with the layering structure of the manufacturing system. This method facilitates cross-view linking and navigation across different perspectives, thereby enriching the system-wide comprehension and utilization of the knowledge graph. However, some limitations typically arise. For instance, the complexity of accurately mapping concepts to appropriate layers can be challenging, especially in dynamic and evolving manufacturing environments. Additionally, maintaining the integrity and consistency of the knowledge graph across different layers can become difficult, potentially leading to misalignment or fragmentation in understanding.

### 5.2 Knowledge graph modeling and population strategy

In order to ensure manageability and scalability, it is crucial to adopt a strategic approach when modeling and incorporating data into KGs. Instead of incorporating all available data, the KG should facilitate an agile, use case-driven approach toward data integration that grounds decisions about what data to integrate into the KG in use-case requirements. Consequently, H-AI systems KGs will not include materialized collections of, e.g., large-scale structured source data that is not graph-structured and can be managed more efficiently in e.g., relational systems or data lakes. For such data, the KG can act as a data catalog that facilitates semantic localization of granular source data for the use case scenarios.

Based on these principles, we propose the following inclusion criteria for data to be added to the H-AI system KGs: (1) Include domain knowledge that can contextualize observational data and/or facilitates automated interpretation (e.g., through rule-based decision support components). (2) Incorporate data that is relevant for the construction of teaming events, i.e., data that affects the execution flow in the teaming process model. For example, *visual quality inspection data (OK/NOK, defect characterization, etc.)* that may trigger teaming processes should be included (e.g., *as quality inspection events*). (3) Include observational knowledge that provides context to other events (e.g., environmental sensor data that qualifies other observations). (4) Incorporate data needed for stakeholder coordination, i.e., data that establishes the context so that the KG can serve as a coordination artifact. (5) Include data leveraged by relational machine learning components, i.e., to enable them to learn, generate insight and make informed decisions based on the KGs. (6) Incorporate data used to construct facts that can be checked against policies, i.e., to ensure compliance with predefined rules and regulations.

Use case-relevant data that does not fulfilll these criteria (e.g., large-scale raw data without well-defined semantics stored in Data Lakes) can be inventoried in the data catalog. Therefore, it is then considered outside the scope of the H-AI systems but may be accessed exogenously by activities that are coordinated through it.

#### 5.2.1 Guidelines for knowledge graph population

To address the challenges regarding knowledge population described in Section 3.2, we propose a set of guidelines and requirements for the KG population in the context of H-AI collaboration: (G1) Knowledge management and integration: The KG needs to integrate heterogeneous data sources and modalities to create a shared understanding among stakeholders and AI systems. It should provide a flexible, graph-based representation of real-world entities, their interrelations, and various topical domains. For example, the KG should describe products, production assets, organizational units, processes, events and activities models. (G2) Stakeholder coordination: The KG should enable collaboration between human agents and AI components by representing a shared mental model and acting as a coordination artifact. This coordination facilitates dynamic population, where the KG dynamically coordinates various agents in tasks such as diagnostic analyses. (G3) Application services: The KG should provide additional capabilities beyond knowledge representation, such as entity resolution, entity linking, and KG completion. Dynamic updating of embeddings in the knowledge engine allows for continuous population and supports downstream application services like predictive services and anomaly detection. (G4) Integrated digital shadow: The KG should accommodate a digital semantic shadow of production assets, capturing their variable properties across their lifecycle. It can link and integrate multiple digital shadows to provide an integrated semantic shadow. The continuous population is essential to maintain the digital shadow with incoming events and data from the production environment. (G5) Dynamic population workflows: The KG should support dynamic extension through collaborative editing by multiple agents. This includes manual curation by domain experts, logical inference by reasoning agents, and statistical inference by machine learning agents.

#### 5.2.2 KG population in the H-AI system architecture

KG population within H-AI System is viewed as a dynamic digital shadow of a production system, incorporating domain knowledge, real-time data, human input, and AI-generated enrichment. It involves automated, semi-automated, and manual activities scheduled or triggered by events, carried out by both human stakeholders and AI agents. Treating KG population as an explicit teaming process addresses the requirements of knowledge management, stakeholder coordination, and integrated digital shadow.

To implement this teaming-centric approach, KG population components (e.g., ETL scripts) should provide APIs for invocation by the teaming engine. This allows flexibility and easy adaptation of KG population workflows by teaming engineers, who can consider them as separate tasks or part of user stories. Not all data in all use cases need to be incorporated through teaming-based population activities, but wrapping population components as services accessible by the teaming engine can maximize flexibility.

**Figure 4:**
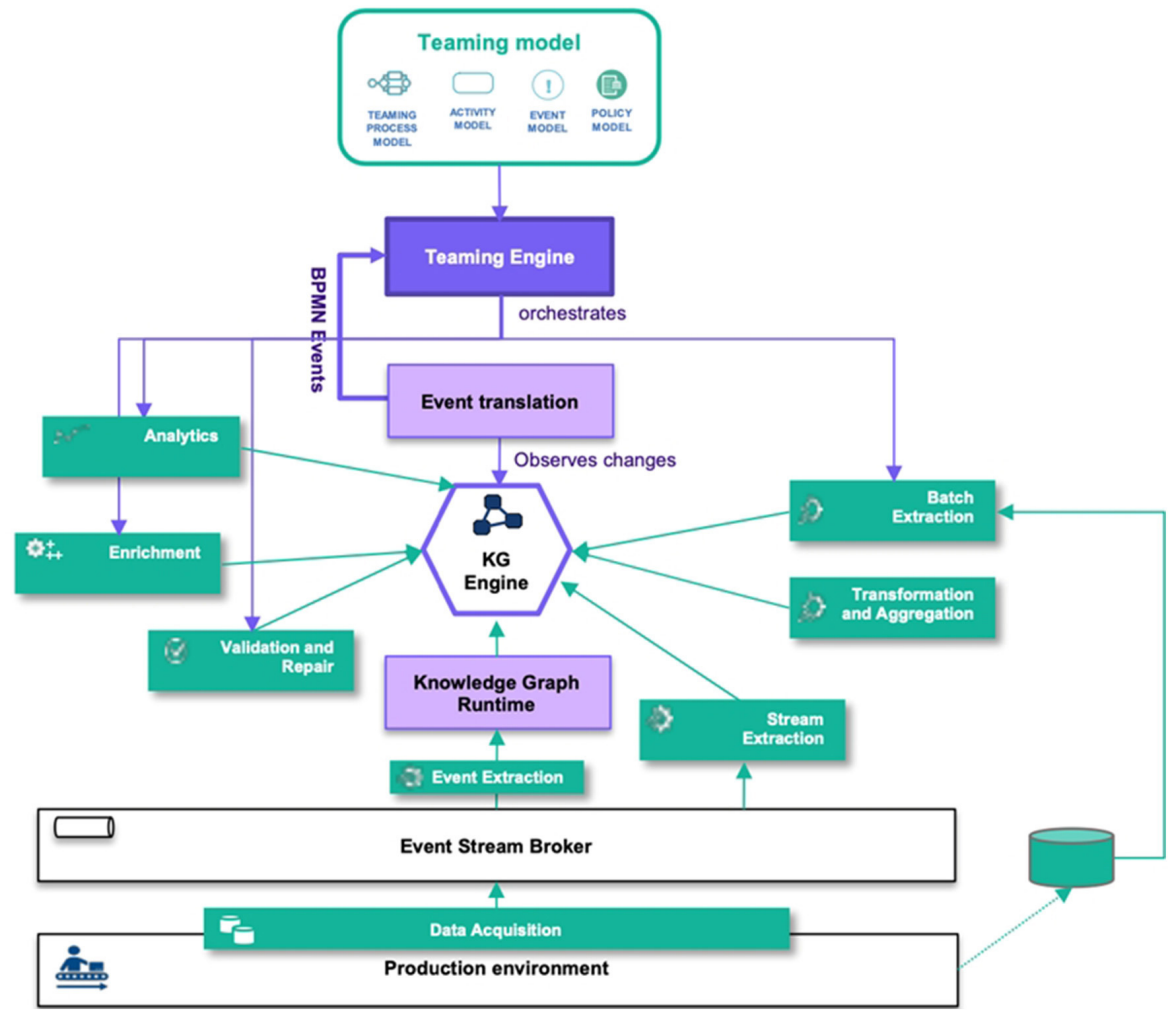
Knowledge graph population in H-AI system architecture.

We proposed a H-AI system architecture that integrates KG population components as a teaming process. Figure 4 shows how the KG population components interact with the H-AI system component. In the following, we discuss the role of each of these platform components for KG population. (i) *Teaming Engine*: The Teaming Engine orchestrates the teaming model, including the teaming process, activity model, event model, and policy model. In the teaming-centric population approach, KG population workflows are considered as teaming processes and KG population activities are treated as teaming activities. The execution flow of the teaming model determines when population activities are performed. KG population tasks can be triggered based on the completion of other activities, scheduled tasks, or teaming events received via the event translation component. (ii) *Event Translation*: The event translation component, facilitated by a synchronization assistant, maps changes in the KG to teaming events. It filters, aggregates, and maps runtime events to teaming events that can be processed by the Teaming Engine. The event translation server does not modify the KG directly but triggers population workflows by mapping runtime events to teaming events. (iii) *Knowledge Graph Engine*: The knowledge graph engine provides a triple-store, query engine, synchronization service, and API. It serves as the core component for managing the KG. The triple-store server stores the KG and offers a query engine with an API. The synchronization assistant detects changes in the KG and facilitates synchronization with other components. (iv) *Knowledge Graph Runtime*: The knowledge graph runtime consumes event streams from the shop floor, lifts, filters, and aggregates raw events into well-defined runtime events, adding observational knowledge to the KG. The KG runtime continuously inserts new triples into the KG based on event data from the shop floors as well as human feedback from the Human Machine Interface (HMI).

#### 5.2.3 Lessons learned

In a H-AI system, the population of a KG acts as a dynamic digital shadow of the production system, incorporating domain knowledge, real-time data, and H-AI interactions. Treating KG population as a collaborative teaming process facilitates effective knowledge management, stakeholder coordination, and the creation of an integrated digital shadow. Nevertheless, there are potential limitations to consider. One challenge is ensuring the continuous and accurate integration of real-time data, which can be affected by data quality issues or system inconsistencies. Additionally, coordinating among multiple stakeholders with diverse expertise and interests might lead to communication barriers or conflicts, complicating the knowledge management process.

### 5.3 Teaming modeling and orchestration

To address the challenge of teaming orchestration described in Section 3.4, we follow a model-based approach that represents H-AI interaction patterns in models, using BPMN as an enabling technology. The resulting teaming process model can then be executed by the teaming engine, a core component of the Teaming.AI software platform. Teaming policies bring dynamics into BPMN-based process executions and thereby allow for late shaping of execution at runtime.

**Figure 5:**
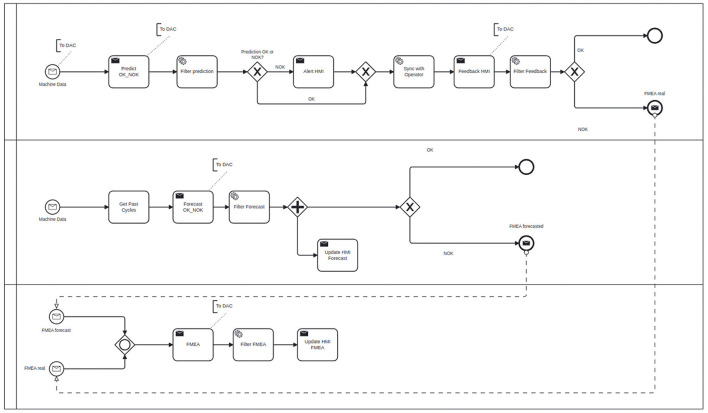
BPMN process model for Scenario 2 example.

As an example, Figure 5 depicts a simplified BPMN process model for Scenario 2 described in Section 2.2.

During the initialization phase, which makes the software platform ready for operation on a concrete use case, teaming engineers and domain experts need to instantiate and customize these teaming process models and integrate it with the target system. Tasks can be instantiated, connected and configured, which then are linked to activities, events, and policies. The use of domain-specific modeling descriptions tailored to the requirements of the respective domain enables efficient and lightweight teaming model development by different domain experts. Model transformations can be implemented to connect the process-oriented view with the information-centric model of the KG and the event-oriented application platform integration. In formalizing the description of H-AI teaming in a teaming process model, recurring elements and integration patterns emerge that reflect different aspects which are necessary or important for the orchestration of H-AI teaming processes, e.g., policy checking, data logging for auditability, and so on.

In addition to the BPMN-based teaming process model, we also define policy, activity, and event models. In practice, this separation enables individual parts to be developed by different domain experts. For instance, a process expert can model the BPMN process, but might not be an expert on how to link it to runtime events. Similarly, legal and ethics policy experts are typically not concerned with technical aspects of data collection for verifying these policies.

To execute the teaming process model at runtime, we leverage state-of-the-art technology. The teaming engine (see also Figure 4) employs both Camunda^3^ (Lammert and Boer, 2015) and Apache Kafka (Garg, 2013) to help in process automation and facilitate synchronizing the information flow. Camunda is an open-source platform for workflow and decision automation. It allows the users to model, automate, and optimize business processes using the BPMN and Decision Model and Notation (DMN) standards. Apache Kafka is a distributed streaming platform that is able to handle high-volume and real-time data streams. It allows real-time data pipelines and streaming applications. By combining both platforms into the core part of teaming engine we allow for data ingestion, processing, and integration across multiple systems and bridge the gap between machine learning and end-users and facilitate process orchestration.

Moreover, the software architecture follows an event-driven communication paradigm, where process workflows are triggered by events. This allows for loose coupling between components and the ability to scale platform processes independently. By using Kafka event streams, the teaming platform can monitor streams of data from various sources and process it in real-time. The processed data can then be used to feed machine learning models or inform decisions within Camunda workflows. Also, ML models can be integrated into Camunda workflows by exposing them as service endpoints. These models can then be used to make predictions or recommendations based on real-time data processing by Kafka.

#### 5.3.1 Lessons learned

BPMN-based teaming process modeling and execution is suited for capturing and orchestrating H-AI teaming workflows. By providing modular teaming activities which may be switched dynamically during runtime using policy constraints, we are able to take into account changing circumstances at runtime. This approach brings more flexibility to process execution by allowing to apply the Teaming Engine to difference use cases just by means of configuring a respective Teaming Process Model. The BPMN language provides a domain-specific modeling language suitable for efficient and lightweight teaming model development by different domain experts. The BPMN model provides a high-level view on the process performed collaboratively by humans and AI agents. On a technical level, a BPMN task is executed by triggering microservice calls to different components, e.g. performing ML prediction, or sending a prompt to the HMI. However, the focus of the teaming engine execution should be on orchestrating interactions with the human instead of coordinating all software communication within the platform so as to avoid teaming engine becoming a performance bottleneck.

### 5.4 Semantic representation of human-AI interaction models

Whereas formalisms like BPMN allow for the modeling of H-AI interactions in manufacturing, they lack the ability to enrich process knowledge and link it to existing domain background knowledge within an industrial KG, as outlined in Sections 5.1 and 5.2. To address this limitation, and allow manufacturers to gain a better understanding of their operations and improve process efficiency and effectiveness, we propose to represent process models and instances in KGs. More specifically, by capturing Teaming process models in a KG and linking them to domain knowledge, we aim to enable new insights about H-AI interactions. Based on such new insights, late shaping can be performed to improve the overall quality of the corresponding knowledge representation and the manufacturing processes in general.

To facilitate process representation, BPMN already provides a graphical notation for standardizing organizational processes, but it is not natively available in RDF. Several domain-specific approaches exist to transform BPMN terminology into RDF-based ontologies such as e3-value (Gordijn, 2004) for e-business processes, BPMN4TOSCA (Kopp et al., 2012) for cloud applications, or the PROV ontology (Moreau and Groth, 2013) for modeling provenance information. Moreover, works exist that intend to introduce generalized BPMN components in ontologies (Annane et al., 2019; Rospocher et al., 2014). For instance, the BPMN Based Ontology (BBO) (Annane et al., 2019) defines a set of concepts and properties to identify a BPMN process diagram as a *bbo:FlowElementsContainer* containing a set of *bbo:FlowElements*, such as activities (*bbo:Activity*), events (*bbo:Event*), gateways (*bbo:Gateway*), or sequence flows (*bbo:SequenceFlow*). Human and AI agents are represented as instances of the *bbo:Agent* class. Thus, we can define activities to be performed by a human agent, an AI agent, or both. Moreover, to represent valid sequence flows, H-AI policies can be applied to structure their interactions.

**Figure 6:**
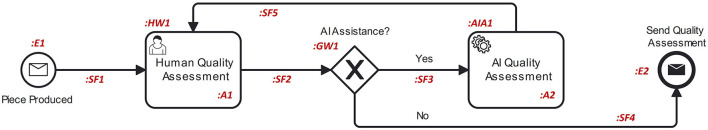
H-AI interaction encoded in a BPMN process diagram, including KG instance URIs.

For instance, the BPMN diagram depicted in Figure 6 represents a manufacturing process that includes a potential human-initiated H-AI interaction. It can be transformed into RDF structures by tools like BPMN2KG (Bachhofner et al., 2022), which is based on the BBO ontology. Thus, we receive KG instances of sequence flows (*SF*), activities (*A*), gateways (*GW*), human workers (*HW*), and AI agents (*AIA*), and events (*E*). These can be enriched with background knowledge, e.g., the required experience of a worker or the underlying ML model(s) of an AI agent. However, this knowledge can only be aligned with process models to further query this information prior to process execution. Although it is possible to define abstract entities of *bbo:Role* or *bbo:Agent* (e.g., *:HW1* and *:AIA1*) that are enabled to perform an actviity, these instances do not represent actual executions of these process components and thus, do not allow for capturing dynamic process executions to subsequently learn from them.

#### 5.4.1 Lessons learned

Several methods exist for capturing BPMN terminology within ontological frameworks and generating KG representations of BPMN process models, these methods typically fall short in incorporating the dynamic executions of these processes. To address this limitation, it is essential to develop solutions that enable the integration of real-time process executions into the KG representations. A potential limitation in this area is the complexity of real-time data integration, which requires robust and flexible data processing mechanisms. Additionally, ensuring that the dynamic aspects of BPMN processes are accurately reflected in the knowledge graph can be challenging, particularly in scenarios with frequent changes or updates to the processes.

### 5.5 Dynamic knowledge graph representations of human-AI interactions

As outlined in the previous section, KGs can be used to represent static BPMN concepts and process models. Based on the information contained within the KG and the current environmental state, subsequent activities can be determined, or agents can be assigned to specific tasks. However, the proposed KG encoding neglects the dynamic aspects of individual process executions. While the dynamic orchestration of production and Teaming processes is implicitly controlled through the information encoded within the KG, the dynamic executions of these processes are not stored in the graph itself. Thus, we aim to analyze approaches for enabling comprehensive representations of process executions, underlying process models, and BPM terminology in a shared KG. By doing so, we do not only highlight the opportunities that arise from integrating dynamic process executions and domain knowledge but also provide insights, guidelines, and implementation approaches for future research in this field. In particular, such formalisms could be used to effectively describe and trace H-AI interaction scenarios.

RDF KGs are typically composed of a concept layer and an instance layer, also referred to as TBox and ABox. While the TBox contains a domain ontology, usually based on the Web Ontology Language (OWL), to define conceptual relationships, the ABox stores the instantiated KG data. In contrast, three abstraction layers are required for describing BPMN process executions, namely the BPMN terminology (such as BBO), the BPMN process model, and the actual BPMN process executions.

While BPMN terminologies need to be stored in the KG TBox and process executions represent ABox instances, the positioning of BPMN process models as process blueprints within the RDF abstraction layers remains ambiguous as they may be regarded both as instantiations of BPMN concepts and conceptual models for BPMN executions. Therefore, we address approaches to integrate BPMN process models either in (i) the concept layer or (ii) the instance layer of a process-enabled KG by applying the idea of (i) OWL constraints or (ii) definite and indefinite instances, respectively. We reuse the BPMN diagram and corresponding H-AI interaction scenario from Figure 6. Furthermore, to showcase the expressiveness of the respective approaches, we attempt to implement the following constraints to further extend this

1. An instance of the AI agent *:AIA1* must utilize one of the pre-trained ML models *:MLX* or *:MLY* (we assume the predicate *:uses_ML_model*).
2. Given an instance of the process *:P0*, only one human isntance *:HW1* may participate in the process instance, i.e., the worker requesting an AI quality assessment also needs to incorporate it.

#### 5.5.1 Representing BPMN process models via OWL constraints

BPMN ontologies like BBO provide a structured framework for capturing BPMN process models within a KG. This approach allows for the representation of process models in the KG TBox, enabling subsequent instantiations in its ABox based on actual process executions. By leveraging this methodology, it becomes possible to encode BPMN process models, such as *:P0*, as subclasses of the *bbo:Process* concept, while specific process elements like the gateway *:GW1* can be represented as a subclass of *bbo:Gateway*.

To ensure the consistency and integrity of the BPMN execution, certain constraints need to be satisfied. For example, it is essential that each instance of *:GW1* is contained within an execution of the process *:P0*. Furthermore, *:GW1* must be linked to an incoming *bbo:SequenceFlow* of type *:SF1* and have exactly one outgoing *bbo:SequenceFlow*, which can be of type *:SF3* or *:SF4*. These execution constraints can be effectively defined using OWL class descriptions and axioms via the equivalent class of *:GW1*


$$\begin{array}{c} (bbo:has\_container\;\text{exactly}\;1:P0)\;\text{and}\;(bbo:has\_incoming\;\text{exactly}\;1\\ :SF2)\\ \text{and}\;(bbo:has\_outgoing\;\text{exactly}\;1\;(:SF3\;\text{or}\;:SF4)), \end{array}$$

encapsulating the necessary execution characteristics and behaviors. Analogously, the previously introduced constraint 1 can be integrated via a superclass of *:AIA1*, i.e.,


$$\begin{array}{c} :uses\_ML\_model\;\text{exactly}\;1\;(\{:MLX,\;:MLY\}). \end{array}$$

Hence, the TBox encodings of BPMN process models serve as valuable representations that capture both valid process flows and constraint-based execution policies. These encodings can be further enriched with domain background knowledge, facilitating the establishment of reusability and integrability.

However, it is important to note that OWL lacks the ability to express overlapping constraints, such as constraint 2. It is not possible to define that two executions of the same process element within an identical process must be performed by the same agent. In such cases, more expressive formalisms like SWRL (Semantic Web Rule Language) need to be employed. However, this introduces additional complexity to the ontology, which is known to hinder the adoption of ontology-based process modeling (Corea et al., 2021).

##### 5.5.1.1 Lesson learned

While there are no restrictions regarding the dynamics of BPMN process executions, the same flexibility does not apply to BPMN process models since process flows are captured within OWL constraints. Any updates or modifications to the models may lead to constraint violations in previously performed executions. This highlights the need for careful consideration and management of the model dynamics within the context of ontology-based models of BPMN process models.

#### 5.5.2 Representing BPMN process models via definite and indefinite instances

Besides OWL-based KG constraints, the alignment of BPMN and KG abstraction layers can also be achieved by encoding BPMN models and BPMN executions within a shared ABox. This approach is in line with existing works like BPMN2KG (Bachhofner et al., 2022), which represent BPMN models and components as instances in a KG. By adopting this methodology, process execution rules can be defined using OWL constraints and by utilizing instance-level properties such as node labels or assignments of agent instances to specific activities. For instance, in order to specify the qualifications required to carry out the AI activity *:AIA1* as per constraint 1, SHACL constraints can be utilized to design corresponding node shapes (Knublauch and Kontokostas, 2017). Therefore, this approach enables the representation of process flows through explicit facts, as opposed to relying solely on OWL class restrictions. Analogously, we can fulfill constraint 2 by defining a single executor of the activity *:A1* or by linking multiple instances of *:HW1* via the *owl:sameAs* relationship. However, to accommodate BPMN executions as KG instances, it becomes necessary to augment the ABox with an additional abstraction layer.

We propose an extension of the KG TBox by introducing the class *:IndefiniteInstance* and the property *:definiteInstanceOf* allowing for the identification of executed BPMN components as definite instances of indefinite BPMN components. While no explicit rules are implemented to automatically verify logical correctness, the ABox encodings of process models allow for more flexibility in representing dynamic BPMN models compared to OWL-based BPMN process model encodings. Furthermore, the instance-level representation contributes to simpler TBox structures, which is a crucial aspect for successful KG and ontology implementations in real-world use cases.

##### 5.5.2.1 Lesson learned

The introduction of definite and indefinite instance layers enhances the expressiveness and dynamics of BPMN-KG integrations, enabling the representation of real-world process executions and their associated BPMN components within a coherent framework. However, when it comes to describing complex manufacturing processes, auxiliary frameworks such as Semantic Web Rule Language (SWRL) or Shapes Constraint Language (SHACL) often need to be employed. These auxiliary frameworks provide additional capabilities for capturing intricate rules and constraints that cannot be easily expressed within the standard BPMN-KG structure. However, a potential limitation is the increased complexity in implementing and maintaining these supplementary frameworks, which may require specialized knowledge and resources.

## 6 Example use case implementations

This section presents illustrative example implementations of the H-AI teaming platform in three use cases within the general scenario settings introduced in Section 2. All use case implementations apply previous findings regarding vertical knowledge integration (Section 5.1), KG modeling and population (Section 5.2), teaming modeling and orchestration (Section 5.3), semantic representation of H-AI interaction models (Section 5.4), and dynamic KG representations of H-AI interactions (Section 5.5).

### 6.1 Use case 1: collaborative quality inspection

As an example of human oversight scenarios (cf. Section 2.1), we implemented a use case where human expertise and flexibility can be integrated with AI-based capabilities in order to optimize quality management in the context of an injection molding production line. In this solution, oversight and decision-making still remain in the hands of human operators, while AI provides decision support. Interaction takes place in a structured and orchestrated manner. The teaming process model and execution may orchestrate human operators and AI agents to collaborate in quality inspection tasks. See also Figure 6 for reference. At production runtime, an AI-based computer vision agent may perform initial quality checks and the teaming engine may request a human operator (via the operator HMI) to double-check in cases of not-OK parts or when the AI is faced with uncertainty or low confidence. Operators have the ability to provide feedback on the quality of AI suggestions, thus realizing closed-loop communication. By incorporating this additional information, the AI agents can continuously learn from human expertise and improve the accuracy and reliability of their predictions.

We apply the guidelines for populating the KG outlined in Section 5.2: (G1) highlights the need for knowledge management and integration, including data from the AI-based quality inspection, human quality inspection, human feedback via the HMI, machine parameter setup, and process model. Stakeholder coordination (G2) involves providing a shared mental model between humans (i.e. quality engineers) and AI agents (i.e., AI-based quality inspectors) to coordinate their tasks while performing collaborative (automated and manual) quality inspections. The application service involves collaborative quality inspection process by taking into account the inspection outcomes (e.g., AI prediction including their level of confidence) and human interpretation and decision (e.g., based on their experience). The integrated digital shadow (G4) is done through the transformation of the involved domain knowledge (e.g., process models, machine information, AI-models, and human feedback) into the KG. The dynamic population workflow (G5) involves manual curation of knowledge sources as well as a population from the dynamic learning of AI-prediction with human feedback when vetoing the decisions.

### 6.2 Use case 2: hybrid H-AI parameter optimization in injection molding

As a use case within machine diagnostics (cf. Section 2.2), we implemented a collaborative H-AI teaming process at another industrial partner from the automotive sector. For this use case, we envisioned a hybrid approach that allows human experts to share their knowledge among each other and with AI agents. In particular, the use case combines the representation of root cause analysis knowledge with the analytic capabilities of ML models to guide the diagnostic process. To this end, we formalize the relevant domain knowledge to coordinate diagnostic tasks and extend them to capture domain knowledge and observed regularities by the stakeholders in a synergistic manner.

**Figure 7:**
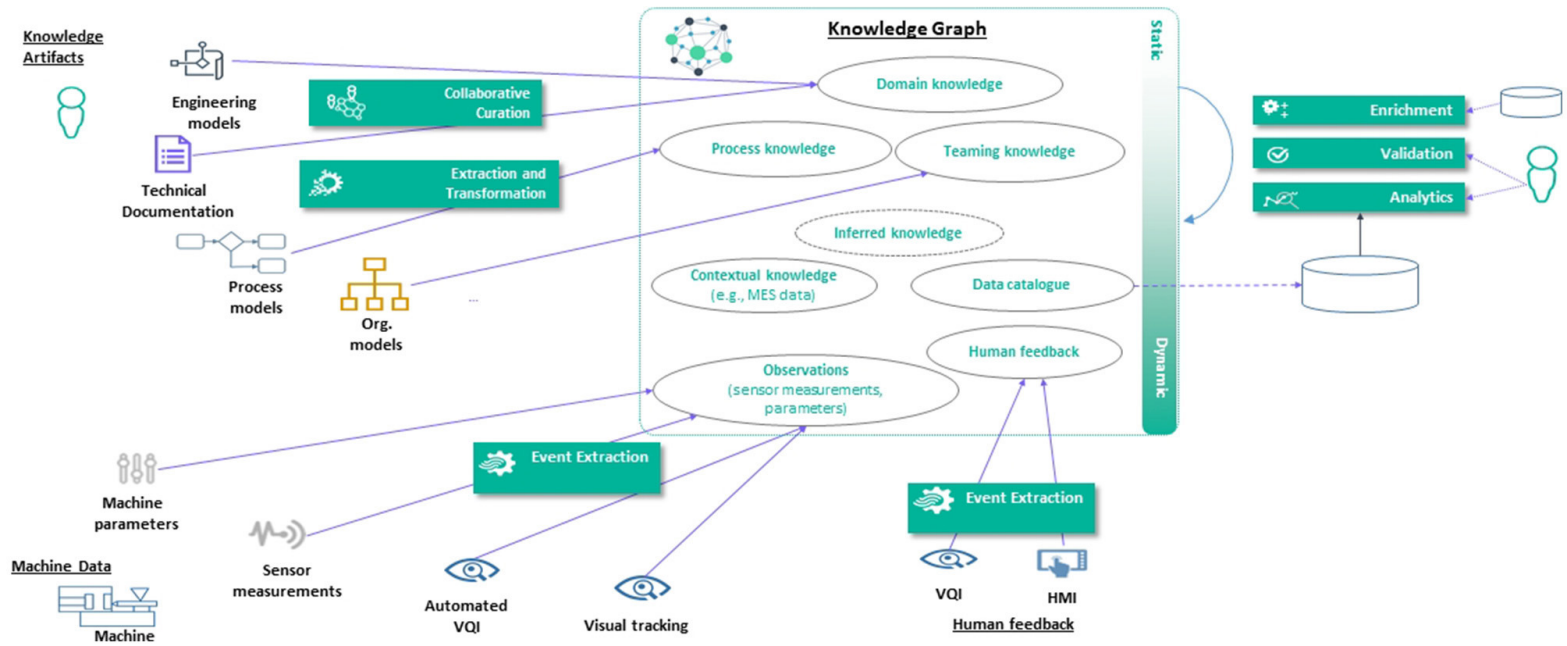
KG population for Use Case 2.

To populate a KG for this use case, we follow the process discussed in Section 5.2. This includes (i) managing and integrating multiple knowledge sources such as injection machine and its parameters, the visual quality inspection system, the HMI, process model, Injection Parameter Adjustment Protocols (IPAP) and Failure Mode and Effect Analysis (FMEA); (ii) Stakeholder coordination focuses on a shared knowledge base between the process model, IPAP and FMEA stakeholders for root cause analysis; (iii) Application services involve optimizing injection parameters by integrating IPAP and FMEA data; (iv) The integrated digital shadow emphasizes the unidirectional information flow indicating that IPAP and FMEA documents are transformed into a KG within the pipeline. (v) The dynamic population workflows involves manual curation of knowledge sources, as well as population from inference services, logic-based or machine learning-based methods. Figure 7 illustrates the KG population system for this use case that facilitate broader tasks e.g., defect prediction, parameter recommendation, failure pattern analysis etc.

### 6.3 Use case 3: occupational risk monitoring and continuous feedback

As a final use case implemented at an industrial partner producing windmill components, we focused on occupational safety and health during hazardous tasks such as industrial milling of large metal parts. In this use case, it is necessary to analyze and predict ergonomic risks on the machine operator. To this end, we dynamically integrate heterogeneous data and knowledge from diverse sources such as monitoring sensors (e.g., the detected objects and poses), production planning systems, process information, rule sets for the assessment of ergonomic risks etc. This requires a trustworthy knowledge base for stakeholders on the shop floor (operators), production planners, process engineers, ergonomic assessors etc. AI agents—including visual tracking systems, objection and pose recognition components, ML-and logic-based inference components for risk scoring purposes over sensor data, e.g., using the Rapid Entire Body Assessment (REBA) assessment method (Hignett and McAtamney, 2000)—can leverage the knowledge base for coordination.

In the implemented use case, high-definition cameras monitor the postures and movements of workers during the whole work shift and the system automatically analyzes it for MSD risks. These risks are reported to the shift managers, who can discuss the information during their daily improvement meetings. With this, the AI is becoming a team member providing important OSH information to the team. The system is set up to improve its analysis on the human input for the next time.

Following the same guideline introduced in Section 5.2, the KG population focuses on addressing the requirement for integrated ergonomic risk assessment. (G1) emphasizes the need for knowledge management and integration, including data from monitoring sensors, process information, and assessment rule knowledge e.g., REBA. Stakeholder coordination (G2) is a key aspect in this use case as they require a combination across multiple heterogeneous sources e.g., operators from the shop floor, production planners, process engineers, and ergonomic assessors. The application services (G3) emphasize the objective of predicting ergonomic risks for machine operators and the integration of heterogeneous knowledge sources to enable a holistic assessment. The Integrated digital shadow (G4) provides the unidirectional flow of data from sources to the KG through a population pipeline e.g., the transformation of the detection data produced by the camera and several other information into a KG. Lastly, (G5) involves the importance of dynamic population workflows involving logic-based inference and human curation of the information sources to score poses and apply assessment rules e.g., REBA assessment method.

## 7 Late shaping of domain knowledge via dynamic KG embeddings

The solution approaches for semantic representations of teaming processes introduced in Section 5 already allow for rule-based late shaping formalisms of domain knowledge based on updates of KGs or process models. Contrarily, in this section we intend to analyze the application of KG embeddings and relational ML models to empirically identify the need for late shapings within our domain. Such methods can be considered since the previously described approaches for knowledge and process modeling are in line with requirements regarding the application of industrial KG embeddings, which were discussed in Section 4.3.

However, traditional KG embedding algorithms focus on capturing stationary graph snapshots and their inherent relationships. However, in real-world scenarios like H-AI teaming, KGs typically need to be regarded as dynamic, with entities and edges being added, modified, or deleted over time. Therefore, it is crucial to develop techniques that can capture the domain dynamics and update KG embeddings accordingly, preferably in an autonomous and real-time manner.

### 7.1 The knowledge engine: implementing dynamic knowledge graph embeddings

Most of the existing works on dynamic graph embedding algorithms do not account for directed and labeled graphs, such as KGs. Rather, they are restricted to undirected and/or unlabeled graphs (such as Pareja et al., 2020; Trivedi et al., 2019; Chen et al., 2021), or they aim to embed temporally enhanced snapshots of stationary graphs (Xu et al., 2020; Dasgupta et al., 2018; Liao et al., 2021). Moreover, approaches like the one proposed in Wewer et al. (2021) exist that intend to concentrate the online training process for updated KG embedding to regions of the graph which were affected by KG updates. However, the overall embedding structure is still affected, leading to a need for continuous adjustments of downstream tasks based on KG embeddings, such as graph-based ML models. Thus, we require a dynamic KG embedding formalism that (i) can produce real-time embeddings for dynamic KGs and (ii) is able to preserve the original structure of KG embeddings to allow for consistent downstream applications.

**Figure 8:**
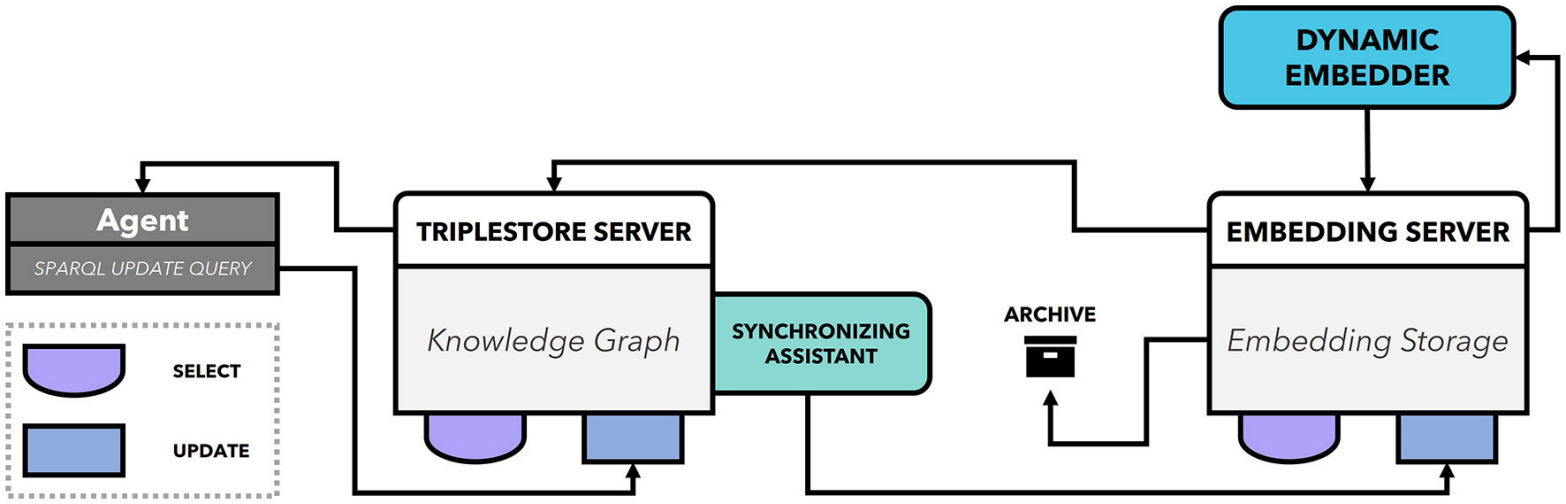
Architectural overview of the proposed knowledge engine methodology.

The proposed method addresses this challenge by introducing the concept of a knowledge engine as illustrated in Figure 8. It intends to extend an existing triplestore framework such as Apache Jena^4^ by means of a KG embedding storage, containing dynamic KG embeddings that is automatically adapted to evolving environments. The dynamic embedder is informed about KG updates through an auxiliary synchronizing assistant which is capable of transforming highly complex SPARQL update queries into sets of inserted and deleted facts. These are archived by the embedding server, including respective timestamps.

At an initial timestamp *t*_0_, the embedding server computes an initial embedding ${\tilde{\gamma }}_{t_{0}}:V_{t_{0}}\to {\mathbb{R}}^{d}$ of the KG $G_{t_{0}}=\left(V_{t_{0}},E_{t_{0}}\right)$ as per Section 4.3. Accordingly, a dynamic KG is defined as a family of stationary snapshots ${\left(G_{t}\right)}_{t\in T}$ with respect to some time set T. Given a *t*>*t*_0_, the dynamic embedder should provide a consistent embedding $\gamma_{t}:V_{t}\to {\mathbb{R}}^{d}$ so that previously trained downstream applications can still be used. To align with these requirements, we introduce the Navi approach as a dynamic KG embedding formalism.

### 7.2 The navi approach: dynamic KG embeddings via local embedding reconstructions

The Navi approach is based on the core idea of GNNs, i.e., to propagate information between connected nodes to capture local and global semantic relations (Krause, 2022). Regarding a node embedding γ(*v*), it leverages the idea to reconstruct it based on its neighborhood embeddings and thus, embedding reconstructions are based on the unique adjacency tensors ${\left(A\left(t\right)\right)}_{t\in T}$ with $A\left(t\right)\in {\mathbb{R}}^{k\times n_{t}\times n_{t}}$. Here, *n*_*t*_ = |⋃_τ ≤ *t*_*V*_τ_| denotes the number of nodes that were known to exist since *t*_0_ and thus, *n*_*t*_≥*n*_*t*_0__ holds.

An initial embedding matrix ${\tilde{X}}_{t_{0}}\in {\mathbb{R}}^{n_{t_{0}}\times d}$, containing initial embeddings as per ${\tilde{\gamma }}_{t_{0}}$. Considering the extended adjacency tensor $\hat{A}\left(t_{0}\right)$, this matrix is reconstructed based on itself via


$$\begin{array}{c} \sigma \left(\hat{A}{\left(t_{0}\right)}_{self}\cdot \Theta_{t_{0}}\cdot W_{self}+\sum_{h=1}^{k}\hat{A}{\left(t_{0}\right)}_{h}\cdot {\tilde{X}}_{t_{0}}\cdot W_{h}\right)\\ =:X_{t_{0}}\approx {\tilde{X}}_{t_{0}}. \end{array}$$

During training, a global embedding $\gamma_{self}\in {\mathbb{R}}^{d}$ is introduced, enabling independence of initial and reconstructed embeddings. It is utilized in self-relations, i.e., $\Theta_{t_{0}}\in {\mathbb{R}}^{n_{t_{0}}\times d}$ contains *n*_*t*_0__ copies of γ_*self*_ and for self-loops in general. Moreover, we randomly replace input node embeddings with γ_*self*_ to simulate the semantic impact of unknown nodes to prevent overfitting. A detailed overview, including benchmark evaluation results, can be found in Krause (2022). The evaluation implies that, given a timestamp *t*>*t*_0_, this approach allows for high-qualitative and consistent embeddings $\gamma_{t}:V_{t}\to {\mathbb{R}}^{d}$ that are computed via


$$\begin{array}{l} \sigma \left(\hat{A}{\left(t\right)}_{self}\cdot \Gamma_{t}\cdot W_{self}+\sum_{h=1}^{k}\hat{A}{\left(t\right)}_{h}\cdot {\tilde{X}}_{t}\cdot W_{h}\right)=:X_{t}, \end{array}$$

i.e., the *i*-th row of $X_{t}$ represents γ_*t*_(*v*_*i*_). In the case of new nodes, ${\tilde{X}}_{t}$ and Θ_*t*_ are extensions of ${\tilde{X}}_{t_{0}}$ and Θ_*t*_0__, respectively, by inserting copies of γ_*r*_0__. Moreover, the update of the adjacency tensor can be performed via


$$\begin{array}{l} A{\left(t\right)}_{h}=I{\left(t_{0},t\right)}^{T}\cdot A{\left(t_{0}\right)}_{h}\cdot I\left(t_{0},t\right)+B{\left(t_{0},t\right)}_{h}. \end{array}$$

On the one hand, the matrix $I\left(t_{0},t\right)\in {\left\{0,1\right\}}^{n_{t_{0}}\times n_{t}}$ accounts for newly inserted nodes, i.e.,


$$\begin{array}{l} I{\left(t_{0},t\right)}_{i,j}=1\;\Leftrightarrow \;i=j. \end{array}$$

On the other hand, the update matrices $B{\left(t_{0},t\right)}_{h}\in {\left\{-1,0,1\right\}}^{n_{t}\times n_{t}}$ represent KG updates


$$\begin{array}{c} ℬ{\left(t_{0},t\right)}_{i,j}\\ =\{\begin{array}{ll} 1 & \Leftrightarrow \text{the edge }(v_{i},r_{h},v_{j})\text{ was inserted between }t_{\text{0}}\text{ and }t\\ -1 & \Leftrightarrow \text{the edge }(v_{i},r_{h},v_{j})\text{ was deleted between }t_{\text{0}}\text{ and }t \end{array}. \end{array}$$

After the KG update, the synchronizing assistant (cf. Figure 8) is to provide (i) the number of nodes *n*_*t*_ and (ii) the update tensor $B\left(t_{0},t\right)\in {\left\{-1,0,1\right\}}^{k\times n_{t}\times n_{t}}$. For instance, given an Apache Jena Fuseki KG, logging tools like rdf-delta^5^ can be extended to use them accordingly. Moreover, while we focus on a single update at time t∈T, transitions between arbitrary timestamps can be handled as well, i.e.,


$$\begin{array}{l} A{\left(t^{\prime }\right)}_{h}=I{\left(t,t^{\prime }\right)}^{T}\cdot A{\left(t\right)}_{h}\cdot I\left(t,t^{\prime }\right)+B{\left(t,t^{\prime }\right)}_{h}\text{ for }t_{0}<t<t^{\prime }. \end{array}$$

To summarize, the late shaping of KG embeddings via Navi reconstructions can be highly valuable for dynamic domains like manufacturing and H-AI due to their ability to capture evolving knowledge in lean representations, offering a powerful tool to analyze and reason over the evolving relationships and characteristics of entities in this domain, such as human and AI agents.

## 8 Conclusions

This work has explored the role of AI in facilitating H-AI collaboration, with a specific focus on computational modeling by means of knowledge graphs. To do that, we have introduced the concepts of early shaping and late shaping design principles and examined how humans might contribute to AI systems' adaptation to operating conditions. Future research will further investigate our knowledge graph-based approach and complement it by recently discussed strategies to utilize generative AI methods, e.g. to streamline the process of populating knowledge graphs from semi-structured sources, as proposed in Pan et al. (2024).

## Data Availability

The raw data supporting the conclusions of this article will be made available by the authors, without undue reservation.

## Footnotes

^1^ https://www.teamingai-project.eu/

^2^ https://osha.europa.eu/en/publications/msds-facts-and-figures-overview-prevalence-costs-and-demographics-msds-europe

^3^ https://camunda.com/bpmn/

^4^Apache Software Foundation, 2021. Apache Jena, Available at: https://jena.apache.org/.

^5^ https://afs.github.io/rdf-delta/
